# Supplementary material for: Changes of in-vivo markers of platelet activation during the menstrual cycle in healthy pre-menopausal female individuals
Source: Commun Med (Lond). 2025 Nov 15;5:533. doi: 10.1038/s43856-025-01240-8 (PMC12738807; doi:10.1038/s43856-025-01240-8)
Supplement: Supplementary file 2 — SUPPLEMENTAL MATERIAL [file 43856_2025_1240_MOESM2_ESM.pdf]

Supplementary Materials

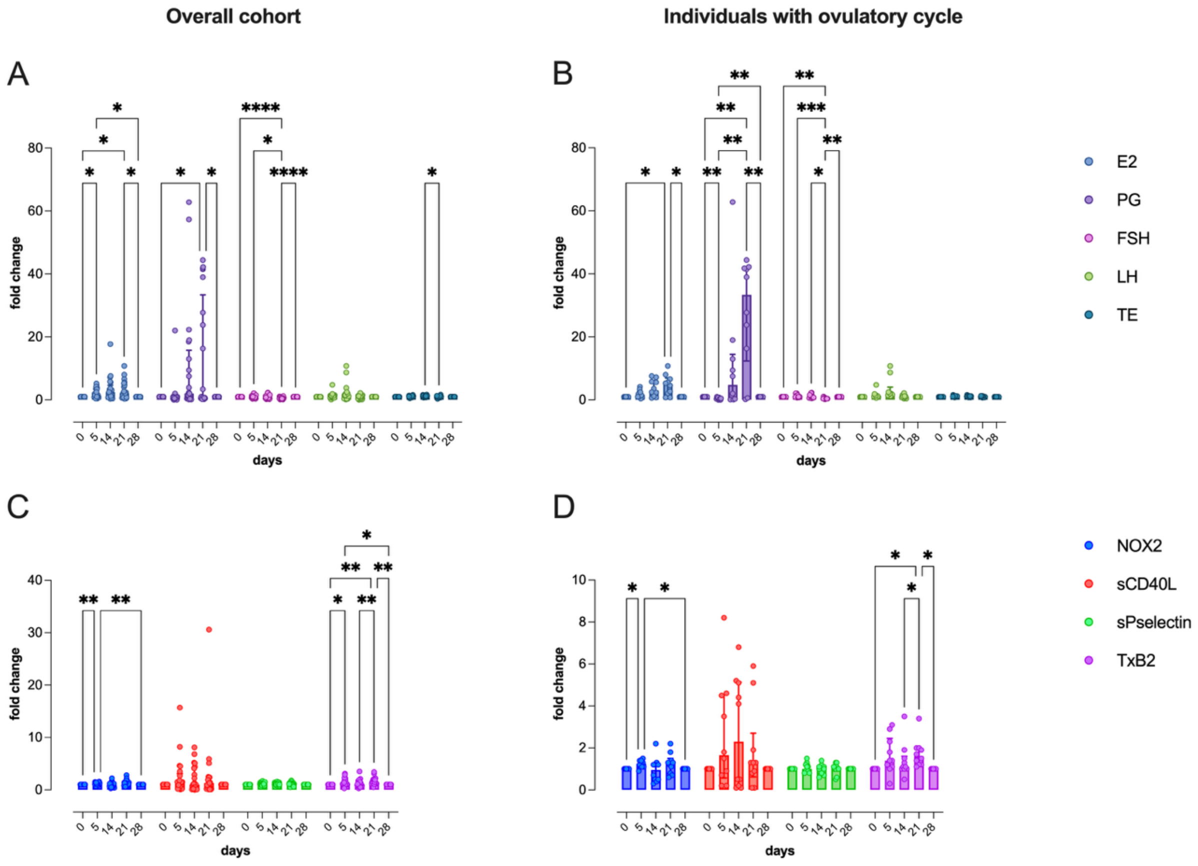

Figure S1. Relative changes of reproductive hormones and in vivo platelet activation markers. Graphs showing the median fold change (relative to the first day of the menstrual cycle) of (A, B) reproductive hormones and of (C, D) the in vivo platelet activation markers among (A, C) the overall cohort (n=21) and (B, D) among individuals with PG≥3ng/ml (ovulatory cycle, n=10). Two-way ANOVA with Tukey's multiple comparisons test were conducted to assess statistical significance. *p<0.05; **p<0.01; ***p<0.001; ****p<0.0001.

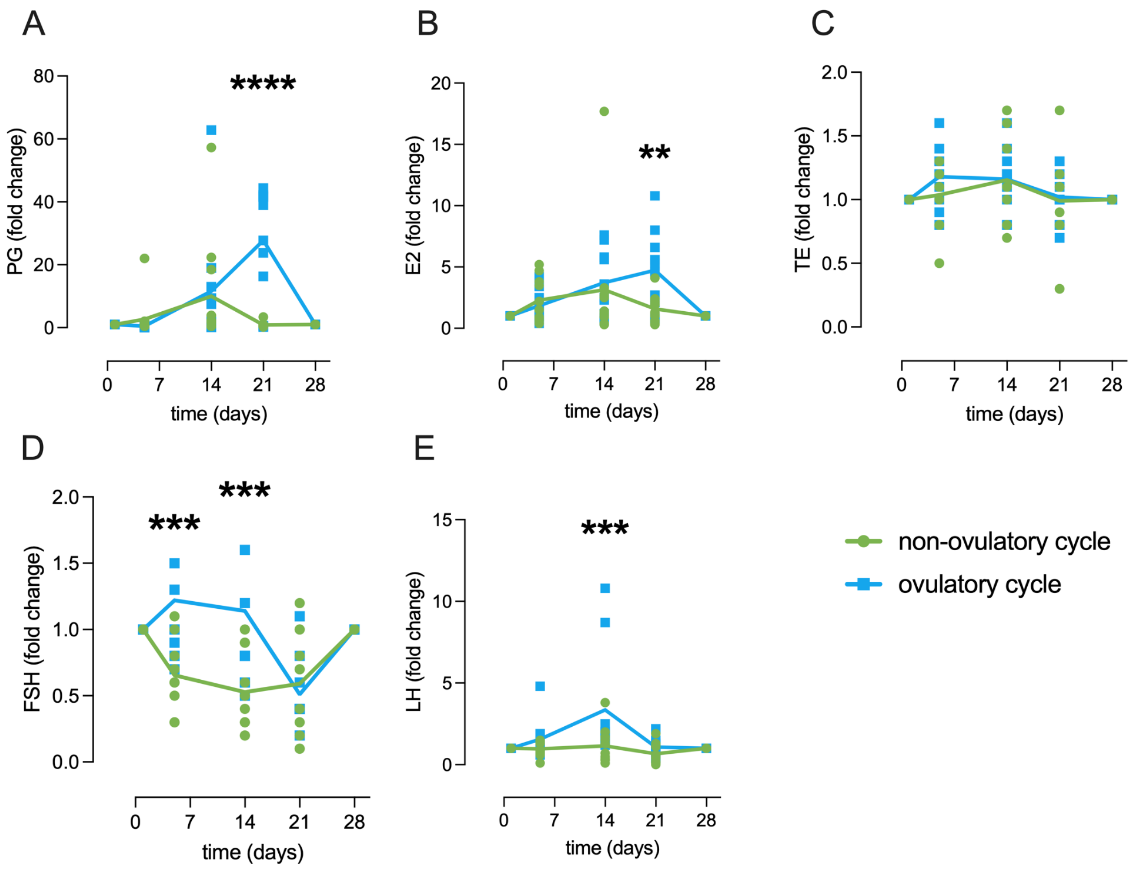

Figure S2. Serum sex hormone levels in individuals with ovulatory and non-ovulatory menstrual cycles. Concentrations of (A) Progesterone (PG), (B) estradiol (E2), (C) testosterone (TE), (D) follicle-stimulating hormone (FSH) and (E) luteinizing hormone (LH) in individuals with PG≥3ng/ml (ovulatory cycle, light blue, n=10) and in individuals with PG<3ng/ml (non-ovulatory cycle, green, n=11). Two-way ANOVA with Sydak's multiple comparisons test were conducted to assess statistical significance. *p<0.05; **p<0.01; ***p<0.001; ****p<0.0001.

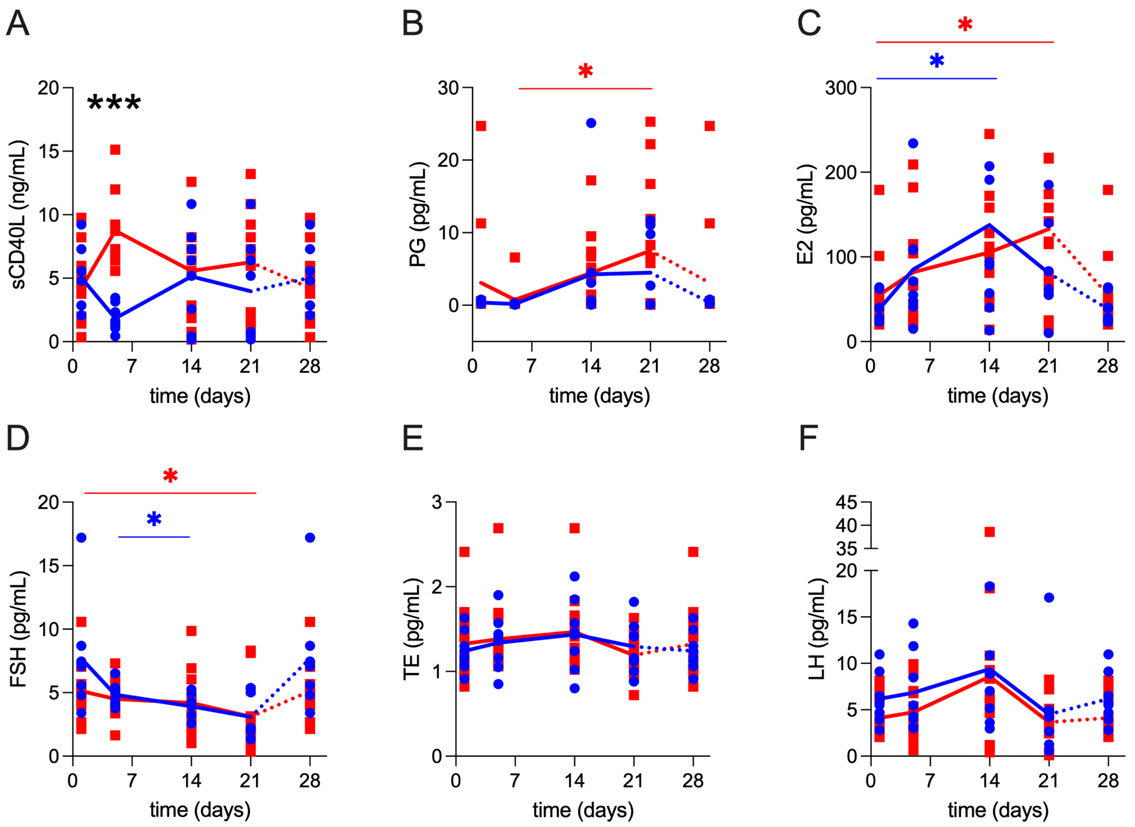

Figure S3. Levels of sCD40L discriminate between women with different hormonal responses. Concentrations of (a) soluble CD40 ligand (sCD40L), (b) Progesterone (PG), (c) estradiol (E2), (d) follicle-stimulating hormone (FSH), (e) testosterone (TE), (f) luteinizing hormone (LH), stratified based on the levels of sCD40L at the end of menstruation. The median and interquartile range is shown in red for those participants that had sCD40L high (n=13) and in blue for those that had sCD40L low (n=8). Ordinary two-way ANOVA test with Šidák multiple comparisons test was used for intergroup (black stars) and intragroup (blue or red) analysis. *p<0.05; **p<0.01; ***p<0.001; ****p<0.0001.

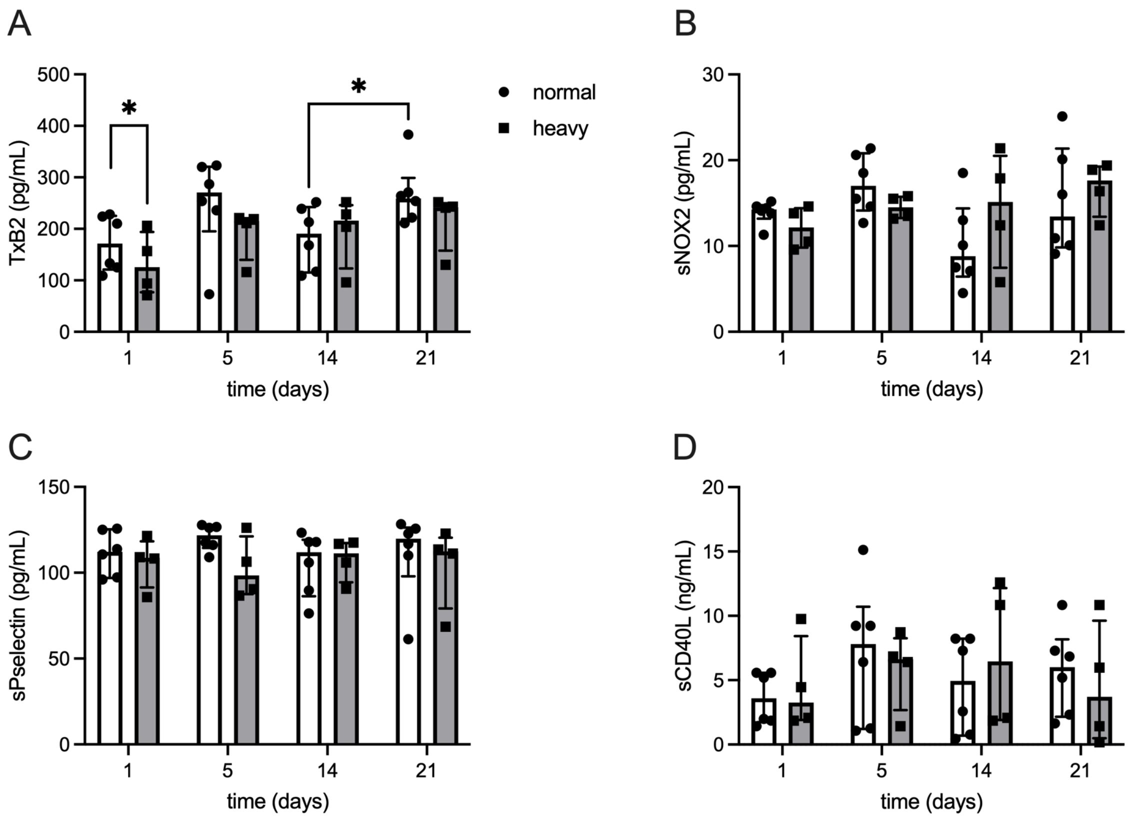

Figure S4. TxB2 associates with heavy menstrual bleeding even when considering only the participants with ovulatory cycle (PG>3ng/ml). Bar graphs of the (a) thromboxane B2 (TxB2), (b) soluble NOX2-derived peptide (sNOX2-dp), (c) soluble P-selectin (sP-selectin) and (d) soluble CD40 ligand (sCD40L) levels stratified based on self-reported heavy (grey with circular dots, n=4) or normal (white with square dots, n=6) bleeding during menstruation. Ordinary two-way ANOVA test with Šidák multiple comparisons test was used for intergroup and intragroup analysis. *p<0.05; **p<0.01; ***p<0.001; ****p<0.0001.

Supplementary Table 1 - Appendix kit and materials

Assay Kit	Company	Reference Code	Sensitivity
Soluble P-selectin (sP-selectin)	Diaclone	850.930.xxx	3.6 ng/ml
Soluble CD40 ligand (sCD40L)	R&D Systems	DCDL40	10.1 pg/mL
Thromboxane B2 (TxB2)	Cusabio	CSB-E08046h	2.34 pg/mL
Soluble NOX2-derived peptide (sNOX2-dp)	Architect System (Abbott Laboratories)	EP3495821A1 (European Patent Office) doi: 10.1111/bcp.13589;	2.87 pg/ml

Supplementary Table 2 - Function and plasmatic range of soluble markers of platelets activation

Platelet markers	Function	Range in plasma of healthy donors mean±SD median [IQR]	References
Soluble P-selectin (sP-selectin)	Soluble P-selectin (sP-selectin) is the ectodomain of the P-selectin receptor. Endothelial cells and platelets store P-selectin in granules in resting conditions. After activation (in the setting of inflammation/vascular injury) it is exposed on the cell surface and subsequently cleaved. Thus, the soluble ectodomain of P-selectin is a marker for platelet and endothelial activation in vivo.	69.3±33.8 ng/mL	[1,2]
Soluble CD40 ligand (sCD40L)	Soluble CD40L is the ectodomain fragment of the CD40 ligand. It is exposed on the surface and cleaved upon platelet activation. Although other immune cells express CD40L, platelets are the primary source of sCD40L in circulation, thus it is considered a marker of platelet activation in vivo.	2.1±0.2 ng/mL*	[3–6]
Thromboxane B2 (TxB2) pg	Thromboxane B2 (TxB2) is a stable metabolite of thromboxane A2 (TxA2), an eicosanoid with para/autocrine function, released by activated platelets to amplify platelet activation and induce vaso-costriction.	152.0 [116.0-187.0] pg/mL*	[7–9]
Soluble NOX2-derived peptide (sNOX2-dp)	sNOX2-dp is the shed ectodomain of the reactive oxygen species (ROS)-producing enzyme NOX2, expressed by platelets and phagocytes (eosinophils, neutrophils, macrophages and dendritic cells). High levels of sNOX2-dp in circulation are associated with platelets activation in sepsis shock and atherosclerosis.	16.3±3.5 pg/mL	[10–13]

*Range calculated only in female donors.

References

1. Ridker, P. M., Buring, J. E. & Rifai, N. Soluble P-selectin and the risk of future cardiovascular events. *Circulation* **103**, 491–495 (2001).
2. Ferroni, P. *et al.* Soluble P-selectin as a marker of in vivo platelet activation. *Clin Chim Acta* **399**, 88–91 (2009).
3. Otterdal, K., Pedersen, T. M. & Solum, N. O. Release of soluble CD40 ligand after platelet activation: Studies on the solubilization phase. *Thromb Res* **114**, 167–177 (2004).
4. Aloui, C. *et al.* The signaling role of CD40 ligand in platelet biology and in platelet component transfusion. *Int J Mol Sci* **15**, 22342–22364 (2014).
5. Danese, S. *et al.* Activated platelets are the source of elevated levels of soluble CD40 ligand in the circulation of inflammatory bowel disease patients. *Gut* **52**, 1435–1441 (2003).
6. Schönbeck, U., Varo, N., Libby, P., Buring, J. & Ridker, P. M. Soluble CD40L and cardiovascular risk in women. *Circulation* **104**, 2266–2268 (2001).
7. FitzGerald, G. A. *et al.* Endogenous biosynthesis of prostacyclin and thromboxane and platelet function during chronic administration of aspirin in man. *J Clin Invest* **71**, 676–688 (1983).
8. Rand, M. L., Reddy, E. C. & Israels, S. J. Laboratory diagnosis of inherited platelet function disorders. *Transfusion and Apheresis Science* **57**, 485–493 (2018).
9. Raparelli, V. *et al.* Testosterone-to-estradiol ratio and platelet thromboxane release in ischemic heart disease: the EVA project. *J Endocrinol Invest* **45**, 1367–1377 (2022).
10. Pastori, D., Pignatelli, P., Carnevale, R. & Violi, F. Nox-2 up-regulation and platelet activation: Novel insights. *Prostaglandins Other Lipid Mediat* **120**, 50–55 (2015).
11. Tiseo, G. *et al.* Interplay between Nox2 Activity and Platelet Activation in Patients with Sepsis and Septic Shock: A Prospective Study. *Oxid Med Cell Longev* **2020**, (2020).
12. Carnevale, R. *et al.* Different degrees of NADPH oxidase 2 regulation and in vivo platelet activation: lesson from chronic granulomatous disease. *J Am Heart Assoc* **3**, (2014).
13. Pignatelli, P. *et al.* Atorvastatin inhibits gp91phox circulating levels in patients with hypercholesterolemia. *Arterioscler Thromb Vasc Biol* **30**, 360–367 (2010).
