# Supplementary material for: Changes of in-vivo markers of platelet activation during the menstrual cycle in healthy pre-menopausal female individuals
Source: Commun Med (Lond). 2025 Nov 15;5:533. doi: 10.1038/s43856-025-01240-8 (PMC12738807; doi:10.1038/s43856-025-01240-8)
Supplement: Supplementary file 3 — Description of Additional Supplementary Files [file 43856_2025_1240_MOESM3_ESM.docx]

**Description of Additional Supplementary Files**

File name: Supplementary Data 1

Description: All data reported in Figures 1, 2, 3, 4, 5, 6 and Supplementary Figures S1, S2, S3, S4
